# Glycerophosphoglycerol, Beta-Alanine, and Pantothenic Acid as Metabolic Companions of Glycolytic Activity and Cell Migration in Breast Cancer Cell Lines

**DOI:** 10.3390/metabo3041084

**Published:** 2013-11-27

**Authors:** Antje Hutschenreuther, Gerd Birkenmeier, Marina Bigl, Knut Krohn, Claudia Birkemeyer

**Affiliations:** 1 Medical Faculty, Institute of Biochemistry, University of Leipzig, Johannisallee 30, 04103 Leipzig, Germany; E-mails: gerd.birkenmeier@medizin.uni-leipzig.de (G.B.); marina.bigl@medizin.uni-leipzig.de (M.B.); 2 Faculty of Chemistry and Mineralogy, Institute of Analytical Chemistry, University of Leipzig, Linnéstr 3, 04103 Leipzig, Germany; E-mail: a_hutschenreuther@eva.mpg.de; 3 University of Leipzig, IZKF Core Unit DNA-Technologies, Liebigstr 21, 04103 Leipzig, Germany; E-mail: krok@medizin.uni-leipzig.de

**Keywords:** breast cancer, metabolite profiling, LDH, Glo1, glycolysis, Warburg hypothesis, cell migration

## Abstract

In cancer research, cell lines are used to explore the molecular basis of the disease as a substitute to tissue biopsies. Breast cancer in particular is a very heterogeneous type of cancer, and different subgroups of cell lines have been established according to their genomic profiles and tumor characteristics. We applied GCMS metabolite profiling to five selected breast cancer cell lines and found this heterogeneity reflected on the metabolite level as well. Metabolite profiles of MCF-7 cells belonging to the luminal gene cluster proved to be more different from those of the basal A cell line JIMT-1 and the basal B cell lines MDA-MB-231, MDA-MB-435, and MDA-MB-436 with only slight differences in the intracellular metabolite pattern. Lactate release into the cultivation medium as an indicator of glycolytic activity was correlated to the metabolite profiles and physiological characteristics of each cell line. In conclusion, pantothenic acid, beta-alanine and glycerophosphoglycerol appeared to be related to the glycolytic activity designated through high lactate release. Other physiological parameters coinciding with glycolytic activity were high glyoxalase 1 (Glo1) and lactate dehydrogenase (LDH) enzyme activity as well as cell migration as an additional important characteristic contributing to the aggressiveness of tumor cells. Metabolite profiles of the cell lines are comparatively discussed with respect to known biomarkers of cancer progression.

## 1. Introduction

Breast cancer, the most common cancer among women worldwide, is known to be a highly heterogeneous disease. It was shown to be distinguishable, for example, according to cell differentiation grade, histological origin, or gene expression profile [1,2,3]. Recent genomic studies classified different subtypes of breast cancer with different prognosis for the clinical outcome [2,4]. However, only few studies about comparative metabolite profiling of breast cancer cell lines are available. Yang *et al.* [5] and Engel *et al.* [6] compared the metabolite profile of a non-tumorigenic with a tumorigenic cell line using GCMS, while Cao *et al.* [7] compared the profiles of the two tumor cell lines, MCF-7 and MDA-MB-231, with a non-tumorigenic cell line, but applied NMR analysis focusing metabolites from choline and phospholipid metabolism. Metabolite profiling has been used in the past as a particularly sensitive screening method to identify various tumor-associated biomarkers in tumor biopsies such as sarcosine, glycine, alanine, *myo*-inositol, lactate and phospholipids [8,9]. Metabolite profiling has also been applied in distinguishing cancer types and in cancer diagnostics [10].

Differential assessment of metabolite profiles from tumor cell lines could be an important tool in cancer research, but is not widely applied. Recent comparative studies of breast cancer cell lines using other methods reported a large variance regarding primary energy metabolism [11]. The glycolytic shift towards aerobic glycolysis in cancer cells in general, referred to as the “Warburg hypothesis” [12] was suggested to be related to increasing aggressiveness of tumor cells and poor prognosis. Consequently, investigation of the Warburg hypothesis has been a major field of cancer cell metabolism research in the past. Isidoro *et al.* [13] showed that the metabolic shift towards glycolysis is also a hallmark of breast cancer.

The glycolytic activity of tumor cells has been connected to changes in mRNA expression of enzymes related to glycolysis such as lactate dehydrogenase (LDH) [13] and glyoxalase 1 (Glo1) [14]. Increased Glo1 expression in particular was described as being related to glycolytic activity in many tumor types [15,16,17]. Glo1 was also recently shown to be related to cancer cell migration and invasion in gastric cancer [18] and Arsenault *et al.* [19] showed that after inhibition of LDH in the breast cancer cell line MDA-MB-435, aerobic glycolysis led to a remodeling of the cytoskeleton facilitating cell migration as another important physiological characteristic of tumor cells and a hallmark of cancer [20].

Based on this, we aimed to differentially assess the known constituents of the molecular phenotype of cancer in cell lines used for cancer research with respect to their known tumor characteristics displayed in Table 1.

**Table 1:** Characterization of breast cancer cell lines for their histological characteristics, cancer specific gene expressions, and tumor aggressiveness.

Parameter	MCF-7	MDA-MB-231	MDA-MB-435	MDA-MB-436	JIMT-1	Reference
Origin	PE	PE	PE	PE	PE	[2,21]
Tumor type	IDC	AC	IDC	IDC	AC	[2,21]
Differentiation grade	High	Poor	Poor	Poor	Poor	[2,21,22]
ER, PR, ERB/B2 status	ER+/PR+	TN	TN	TN	ERB/B2+	[23,24]
p53 status	wt	m	m	m	m	[2,23]
*In vitro* invasiveness	+/ ++	+++/ +++++	+++/ +++	++/ +++	n.a. n.a.	[22] [24]
Tumors in nude mice	P	LI	LI	LI	Yes, not further specified	[21,24]
Gene cluster	luminal	basal B	basal B	basal B	basal A/ ERB/B2	[2,25]

PE = pleural effusion; IDC = Invasive ductal carcinoma; AC = adeno carcinoma; ER = estrogen receptor; PR = progesterone receptor; TN = triple negative; wt = wild type; m = mutated; P = primary tumor; no local invasiveness or metastasis; LI = local invasiveness.

In summary, we present a comprehensive characterization of five widely used breast cancer cell lines, namely MCF-7, MDA-MB-231, MDA-MB-435, MDA-MB-436, and JIMT-1, using biochemical and physiological methods. Within this study, metabolite profiles were related to physiological characteristics of these cell lines such as cell migration and selected enzyme activities. Metabolite patterns were comparatively discussed addressing (i) differences in genetic subgroups of the cell lines (Table 1), (ii) metabolites related to glycolysis and the tricarboxylic acid (TCA) cycle, and (iii) the extent of glycolytic activity by lactate released into the cultivation medium. Though tumor progression and phospholipid metabolism has been related elsewhere [26,27,28], to our best knowledge, this is the first study establishing a link between the glycolytic activity of cancer cell lines, cell migration behavior and the relative abundance of glycerophosphoglycerol (GPG), beta-alanine and pantothenic acid (PA). In addition, cancer markers known from other studies are discussed within the context of the aggressiveness of the investigated breast cancer cell lines.

## 2. Experimental Section

### 2.1. Materials and Chemicals

Reduced glutathione (GSH), protein marker Roti^®^-Mark, Rotiphorese Gel 30 SDS Ultra-Pure and TRIS were from Carl Roth (Karlsruhe, Germany), Coomassie Brilliant Blue R250, *N,N,N’,N’*-tetramethylethylenediamine (TEMED), 3,3-diaminobenzidine-4-hydrochloride (DAB) and β-mercaptoethanol were from Serva (Heidelberg, Germany); RPMI 1640 medium (cat. no. 21875-034), Dulbecco’s modified Eagle medium (DMEM) supplemented with 4.5 g glucose/L (cat. no. 41966-029), Opti-MEM^®^, fetal calf serum (FCS) (cat. no. 10500-064), glutamine (cat. no. 25030-024), penicillin/streptomycin (100 U penicillin/mL; 100 mg streptomycin/mL) (cat. no. 15140-122) and trypsin/ethylenediamine-tetraacetate (EDTA) were from Life Technologies (Darmstadt, Germany), hematoxylin was from Merck (Darmstadt, Germany), milk powder from Heirler Cenovis (Radolfzell, Germany), bovine serum albumin from PAA Laboratories (Linz, Austria). Tumor cell lines used were the human breast cancer cell lines MCF-7 (DSMZ ACC115), MDA-MB-231 (ATCC HTB-26), MDA-MB-435 (DSMZ ACC 65), MDA-MB-436 (ATCC HTB-130), and JIMT-1 (DSMZ ACC 589;). *N*-methyl-*N*-trifluoroacetamide (MSTFA) was purchased from Macherey-Nagel (Düren, Germany), pyridine and methoxyamine hydrochloride were from Fluka (Buchs, Switzerland), methanol was from VWR (Darmstadt, Germany), and trypan blue and phosphate-buffered saline (PBS) from Seromed (Berlin, Germany). All other chemicals were from Sigma-Aldrich (Taufkirchen, Germany) and all additional cell culture material from Greiner Bio-One (Frickenhausen, Germany).

### 2.2. Cell Culture

MCF-7 and JIMT-1 cells were cultured in RPMI 1640 medium, MDA-MB-231, MDA-MB-435 and MDA-MB-436 cells were cultured in DMEM. Medium was always supplemented with 10% fetal calf serum (FCS) and 2 mL glutamine (2 mM). All cells were grown at 37 °C in a humidified atmosphere containing 5% CO_2_ using an incubator (Hera Cell 150 Heraeus, Hanau, Germany). Except for JIMT-1, each cell medium contained penicillin/streptomycin (100 U penicillin/mL; 100 mg streptomycin/mL). Cells were grown in T25 or T75 cell culture flasks, or in 24-well plates depending on the experimental conditions.

Enzymatic cell disruption by trypsinization, and direct extraction of cells from 24-Well plates was carried out according to [29].

Cell count and viability was determined by diluting the cells 1:1 with trypan blue (0.4% w/v in PBS) and counting in a Neubauer counting chamber using a light microscope (ID3 Carl Zeiss, Jena, Germany).

### 2.3. Stable Transfection of MCF-7 Cells with a shRNA-Expressing Plasmid for GLO1 Silencing

Human GLO1 specific shRNA was designed as 63-mer containing a hairpin-loop and cloned into pSuper vector with the H1 RNA polymerase promoter. The vector described in van de Wetering *et al.* [30] with an inducible system for stably integrated siRNA and an EGFP cassette was used. A Zeocin-resistance cassette allowed stably transfected eukaryotic cells to be selected. The oligonucleotides encoding the Glo1-shRNA were shGlo1 Fw: 5′-GATCCCG-CATCTAGGACTGATGGATTTCAAGAGAATCCATCAGTCCTAGATGCTTTTTGGAAA-3′ and shGlo1 Rv: 5′-AGCTTTTCCAAAAAGCATCTAGGACTGATGGATTCTCTTGAAATCCA-TCAGTCCTAGATGCGG-3′. Transfection was conducted using TurboFect^™^ according to the manufacturer’s instruction. Briefly, cells were seeded in 6-well plates and cultured over night until 50%–70% confluence. Cultivation medium was replaced by Opti-MEM and cells were transfected immediately with a mixture of 4 µg DNA in 1 mL Opti-MEM and 6 µL TurboFect^™^ incubated previously for 20 min at room temperature. Cells were incubated 5 h before Opti-MEM medium was replaced by normal cultivation medium. After two days, cells were harvested by trypsinization, split to about 20% confluence and positively transfected cells were selected using Zeocin (250 µg/mL) and EGFP fluorescence. Glo1 protein content and Glo1 enzyme activity were tested in the resulting MCF-7 siGlo1 mutants and mock vector transfected cells.

### 2.4. Metabolite Extraction, Derivatization and GCMS Analysis

Methanolic extraction of metabolites from cell samples, derivatization and GCMS analysis were performed as described previously [29]. Metabolites from the cultivation medium were similarly derivatized using a 10 µL aliquot of vacuum evaporated cultivation medium.

We created two datasets with n = 6 each addressing the issue of biological variance as well as fast turnover of metabolites enhancing the robustness of our results. Thus, we used one trypsinized sample batch (sample set 1) that was consecutively harvested over a time period of 6 weeks (one replicate per week for each cell line). All analyses were performed within each replicate set from the same cell pool (Glo1 enzyme activity, cell migration, protein and mRNA level of Glo1 for the corresponding profiling data). The second sample batch (sample set 2) was directly extracted from the well plate. In this case, analysis of all parameters from the same cell pool was not feasible but extraction allowed for faster quenching of cellular metabolism.

### 2.5. Determination of Medium pH

An aliquot of cells from each cell line was seeded into 24-well plates. After 24 h, the medium was changed, the pH value of the control medium and the pH-value in the cultivation medium of cells at 48 and 72 h, respectively, was determined using a pH-electrode (Spintrode 238197 Hamilton, Hoechst, Germany). The pH decrease in the cell medium at each time point was calculated for each cell line and expressed as the mean difference (n = 6).

### 2.6. Protein Extraction, Determination of Protein Content and Immunoblotting

Cytosolic protein extracts were prepared as described in [31] and protein content of the supernatant was determined in duplicate according to Bradford [32]. For western blot analysis, equal amounts of protein were loaded to SDS-pore gradient gels (4%–20%) and run under reducing conditions. Proteins were blotted to cellulose nitrate membranes (Whatman Schleicher and Schuell, Dassel, Germany) and Glo1 was detected by anti-Glo1 polyclonal antibodies (1 μg/mL; 1:4000 in PBS) (BioMac, Leipzig, Germany) in combination with goat anti-rabbit Ig-HRP (1:4000) (Dianova, Hamburg, Germany). As loading control, β-actin was analyzed using rabbit anti-β-actin Ig (1:4000) (Acris, Hiddenhausen, Germany) in conjunction with HRP-labeled goat anti-rabbit Ig. Bands were visualized using either 1 mM DAB and 0.03% H_2_O_2_ as substrate for HPR reaction, or by chemiluminescence (Thermo Scientific, Rockford, USA) according to the manufacturer’s instructions.

### 2.7. Enzyme Activity Measurements

LDH activity measurement was carried out as described previously [31], Glo1 activity was determined at 240 nm according to [33]. A mixture of 0.2 mM reduced glutathione, 0.2 mM methylglyoxal (MGO) and 50 mM phosphate buffer pH 7.0 was incubated for 2 min before 10 µL cell lysate was added to the reaction mixture resulting in a total volume of 1 mL. Enzyme activities were measured at 25 °C for 3 minas absorbance at 240 nm against a reference without cell lysate. All enzyme activities were expressed in U/mg total protein.

### 2.8. RNA Isolation, Reverse Transcription, Semi-Quantitative and qRT-PCR

Total RNA of different breast cancer cell lines was isolated using TRIzol^®^ reagent. RNA concentration of the pellet resuspended in 50 µL DEPC water (0.1% v/v diethylpyrocarbonate) was determined at 260 and 280 nm using a nanodrop spectrometer ND 1000 (NanoDrop technologies, Wilmington, USA). Reverse transcription of RNA from the different cell lines was performed using GoScriptTM Reverse Transcription System (Promega, Madison, USA) according to the manufacturer’s instructions and 1 µg RNA for each sample. Reaction was performed in a thermal cycler PTC 200 (MJ Research, St. Bruno, Canada) for 1 h at 42 °C and 10 min at 70 °C. cDNA was stored at −80 °C until further use. Semi-quantitative PCR for Glo1 and *β*-actin was performed using GoTaq^®^ Flexi DNA Polymerase (Promega, Madison, USA) according to the manufacturer’s instructions using 5 µL cDNA per sample. The PCR program for Glo1 was as follows: denaturation 5 min at 95 °C, 20 cycles 30 s at 95 °C, 1 min at 68 °C, 1 min at 72 °C and, finally, 5 min at 72 °C using 5'-AGGGCATCAATCAGCTCAAC-3' as forward primer sequence (fw) and 5-CCAAGAGCACAATGGTCAAA-3 as reverse primer sequence (rv), β-actin primer were 5'-AGAAAATCTGGCACCACACC-3' (fw) and 5'-CTCCTTAATGTCACGCACGA-3' (rv). 20 µL PCR product and 8 µl of a 100 bp DNA ladder reference standard (Bioron, Ludwigshafen, Germany) were loaded onto separate lanes on a 1% agarose gel containing ethidium bromide and electrophoresis was run at 100 V for 30 min. GLO1 knockdown in MCF-7 siGlo1 cells was confirmed by qRT-PCR according to the method described in Lindner *et al.* [34]. GLO1 mRNA was quantified by qRT-PCR in the five breast cancer cell lines using a TaqMan Gene Expression Assay with hydroxymethylbilane synthase (HMBS) as housekeeping gene (Applied Biosystems, Foster City, USA) and TaqMan Gene Expression Mastermix (Applied Biosystems, Foster City, USA). Each sample contained 2 µL cDNA in a total volume of 10 µL, incubated at 50 °C for 2 min, at 95 °C for 10 min and 48 cycles at 95 °C for 15 s and at 60 °C for 1 min on a 7500 real time PCR system (Applied Biosystems, Foster City, USA). Data was evaluated with the instruments software SDS 1.4 [35].

### 2.9. Migration Assay

Cell migration was assessed using the Boyden chambers assay according to [34] using a reduced incubation time of 4 h. Cell migration was maximum-normalized within each day and expressed as the mean value of relative migration of five independent experiments.

### 2.10. Sample Evaluation and Statistical Analysis

Peak identification was performed as described before [29], using a customer library with reference mass spectra. For normalization, peak areas were divided by the sum of all peak areas from the corresponding chromatogram. Statistical analysis was performed using MS Excel 2010 (Microsoft, Redmont, Washington, WA, USA) and IBM SPSS Statistics 19 [36]. Unless stated, only metabolites present in 90% of the overall samples with no more than two missing values among six replicates were considered. Missing values were replaced by mean values of the replicate set if necessary. The difference in the sum of all peak areas within a particular chromatogram (here referred to as “total ion current sum, TIC sum”) was tested before normalization to not exceed a factor of two for each sample pair and sample set [29] and cell counts were adapted for the final experiment.

## 3. Results and Discussion

### 3.1. Metabolite Profiles of Cancer Cell Lines are Cell-Type Specific

Metabolite profiles of the five breast cancer cell lines (MCF-7, MDA-MB-231, MDA-MB-435, MDA-MB-436 and JIMT-1) with different origin and different molecular characteristics (Table 1) were compared to each other. The mean relative standard deviation (RSD) over all normalized compound areas for sample set 1 (with interday replication, n = 94) was between 47% for MDA-MB-435 and 64% for MCF-7 with an average of 54% RSD over all cell lines. Sample set 2 based on intraday sample replicates had an RSD ranging from 28% for JIMT-1 cells to 31% for MDA-MB-231 including 117 analytes.

**Figure 1:**
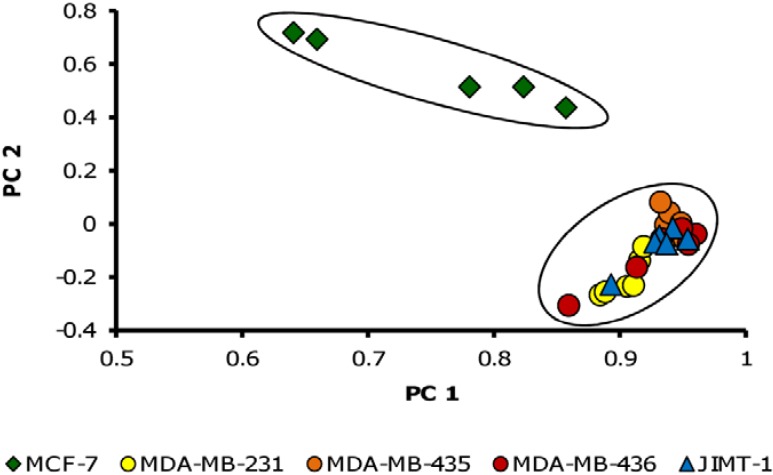
Principal component analysis (PCA) of metabolite profiles from the five breast cancer cell lines. MCF-7 is clearly separated from the other cell lines. PC 1 includes 81% of variability, PC 2 7.5%. n = 6 for each cell line, only metabolites with significant differences between at least two cell lines (*p* < 0.05; mean ratio > 2) were included (n = 33).

All identified metabolites were assessed for significant differences between the replicates of any two cell lines of the data set with Student’s t-test. Using the metabolites with significant differences between at least two cell lines (*p* < 0.05; mean ratio > 2) a principal component analysis (PCA) was performed, shown in Figure 1.

The metabolite profile of MCF-7 cells exhibited the largest difference to the other tested cell lines (illustrated exemplarily for sample set 1). All other cell lines did not separate from each other. The higher variation observed for MCF-7 replicates may be due to the stronger attachment of MCF-7 cells to the cultivation flasks, resulting in a higher variation of the sampling process in comparison to the other cell lines.

All identified analytes that were significantly different between MCF-7 and all other cell lines (Student’s t-test) with the same intensity pattern (signal ratio below or above 1) in both sample batches, are listed in the online resource (see the Supplementary Table S1). We found that most of these metabolites belong to amino acid metabolisms according to the *Kyoto Encyclopedia of Genes and Genomes* (KEGG) [37]. Among the cell lines used in this study, MCF-7 is the only estrogen receptor (ER)- and progesterone receptor (PR)- positive cell line belonging to the luminal gene cluster and exhibiting a high differentiation grade (Table 1). Table 2 lists all metabolites that were significantly different in one cell line compared to all others and were therefore candidate diagnostic markers for the respective cell line. We included only those metabolites with mean ratios > 2 to reduce false positive results according to our previous findings [29].

**Table 2:** Analytes with significantly different concentration in one cell line compared to all other cell lines. Metabolites of sample set 2 had to be significantly different after 24 h and after 48 h with mean ratios above 2 at both time points. Significance was assessed with Student’s t-test. *p* Values of the sample set 2 were calculated for each cell line against all others of the 48 h-value (n = 6; n.d. = not detected).

Cell line	Analyte	Relative abundance	*p*-values sample set 1	*p*-values sample set 2
MCF-7	Cystathione (2TMS)	Increased	n.d.	1.2×10^−6^
	Asparagine DL (3TMS)	Increased	0.05	2.6×10^−4^
	Gulonic acid (6TMS)	Increased	0.37	1.3×10^−3^
	Piperidine-2-one, 3-amino (2TMS)	Increased	n.d.	6.6×10^−3^
	Proline 4-hydroxy-(3TMS)	Increased	0.03	5.8×10^−5^
	Hexadecan-1-ol, n- (1TMS)	Increased	0.02	4.4×10^−4^
	Arginine DL --NH3 (3TMS)	Increased	n.d.	2.6×10^−3^
	Ornithine DL (4TMS)	Increased	0.19	6.6×10^−5^
	Ornithine DL (3TMS)	Increased	n.d.	5.0×10^−3^
	Inositol *myo* (6TMS)	Decreased	6.2×10^-13^	6.6×10^−8^
	Alanine beta (3TMS)	Decreased	2.6×10^-5^	1.7×10^−9^
	Octadecan-1-ol, n- (1TMS)	Increased	0.04	2.4×10^−4^
JIMT-1	Pantothenic acid D (3TMS)	Increased	0.08	1.2×10^−5^
	Alanine (2TMS)	Increased	1.4×10^-4^	5.3×10^−4^
	Glutaric acid, 2-hydroxy (3TMS)	Increased	n.d.	2.1×10^−9^
	Glutamine DL (4TMS)	Decreased	n.d.	9.3×10^−6^

Based on the number of listed metabolites in Table 2, it is apparent that MCF-7 cells are most different from all other tested cell lines. MCF-7 cells have the highest number of significantly different metabolites, followed by JIMT-1. For MDA-MB-231 and MDA-MB-435, no more than one unidentified peak was different in its relative intensity; in MDA-MB-436, proline was the only compound that was significantly decreased compared to all others (not listed). Proline is known to be a non-specific stress responder [38], but was also found to increase through *de novo* synthesis in tumor cells with early metastasis [39]. Higher amounts of proline were detected in tumorigenic MDA-MB-435 cells compared to non-tumorigenic MCF-10A cells [6] and increased proline synthesis was associated with increased turnover of the extracellular matrix of metastatic tumor cells.

Engel *et al.* [6] recently compared profiles of MCF-12A and MCF-7 as epithelial and tumorigenic breast cell cultures, respectively, and found the relative abundances of *N*-acetyl aspartate, lactate, and cystathione increased the most in the tumor cells. However, according to our results cystathione was highest in MCF-7 although MCF-7 is the least tumorigenic cell line. Notably, differences among tumor cell lines according to their tumor characteristics are not necessarily in agreement with a comparison of malignant *vs.* non-malignant cells. Yang *et al.* [5] investigated differences in metabolite profiles of tumorigenic MDA-MB-435 cells and non-tumorigenic MCF-10a cells. They found *myo*-inositol to be increased in the malignant cell line, which was also higher in the more tumorigenic cell lines in our analysis. We further found that the relative intensities of two fatty alcohols (C16 and C18) were much higher in MCF-7 and observed different relative intensities of several amino acids and pantothenic acid (PA) compared with the other cell lines (see the Supplementary Table S1).

All three basal B cell lines had very similar metabolite patterns. Considering these three cell lines as “replicates” of one group, we tested for significant differences against the other two gene cluster type cell lines. Only two metabolites were found to be putative candidate markers for the basal B cell lines in both experiments, namely 4-hydroxyproline (decreased in the basal B cell lines) and 4-aminobutyric acid (GABA, increased). The difference between basal A and basal B was suggested to correspond to the epithelial-mesenchymal transition (EMT) [40]. Gene cluster analysis of breast cancer cells had previously shown the least similarity between the luminal type and the two other types, basal B and basal A/ ERB/B2 [2,41]. The obtained metabolite profiles resembled the extent of reported diversity, and so metabolite profiling may prove useful as an additional tool for characterization or classification of tumor cell types, along with proteomics and transcriptomics. However, only few data about comparative metabolite profiling of breast cancer cell line subtypes are available, usually tumorigenic are compared with non-tumorigenic cell lines. To our knowledge, this is the first time metabolite profiles of more than two breast cancer cell lines with different genetic signature have been compared.

Finally, our data address the issue of the biological variance within datasets and independent replicate measurements in metabolomics research. This becomes obvious when comparing thep-values for sample sets 1 and 2, in which all replicates were harvested the same day; lower p-values were found with sample set 2. In sample set 1, some of the listed metabolites were not detected in enough samples or the differences were not significant. Also, the relative intensities of one metabolite in the five cell lines were sometimes different in sample set 1 and sample set 2. Biological variance was shown before to be about twice the intraday variance [29]. Thus, in order to confirm correlations in metabolic regulation, we strongly recommend incorporating the interday biological variability with independent replicates. (Note: Intraday variance of metabolite profiles generated from directly extracted and trypsinized cells was constant (data not shown), so that we consider the harvesting method itself not crucial for the observed differences within our sample sets.)

### 3.2. Lactate Release Is Enhanced for MDA-MB-231 and JIMT-1

Since breast cancer cells were shown to comply with the Warburg hypothesis [13], we examined metabolites belonging to glycolysis and the TCA cycle in more detail. According to the Warburg hypothesis, tumor aggressiveness is related to the energy metabolism of cells. Consequently, different relative intensities of metabolites belonging to energy metabolism could be expected according to the differential aggressiveness of the analyzed tumor cell lines. Based on the cell line characteristics displayed in Table 1, we would expect a high abundance of glycolytic metabolites and low abundance of TCA cycle metabolites in the poorly differentiated and invasive cell lines, and the opposite in MCF-7 cells. However, contrary to our expectations, metabolites belonging to the TCA cycle or to glycolysis were not among the distinguishing, significantly different metabolites. In sample set 2 only, 1,3-dihydroxyacetone phosphate and 2-oxoglutarate could be additionally detected. Both analytes had significantly increased relative amounts in MDA-MB-231 and JIMT-1 compared to MCF-7 (mean ratio > 2, *p* < 0.05) indicating possible changes in energy metabolism. The relative intensities of these metabolites are listed for all cell lines compared to MCF-7 as a reference in online resource (see the Supplementary Table S2).

It has been previously shown that some cell lines, such as MDA-MB-231 cells, rely much more on aerobic glycolysis than MCF-7 cells [11] and that cancer cells release lactate as an end product of aerobic glycolysis with a high acidification potential. Hori *et al.* [42] found increased lactate in serum of tumor patients, in lung tumor tissue and with tumor progression. Lactate release of tumor cells is thought to be related to the glycolytic shift of cells contributing to tumor development [43]; lactate release would be an indicator of glycolytic activity in cells.

**Figure 2:**
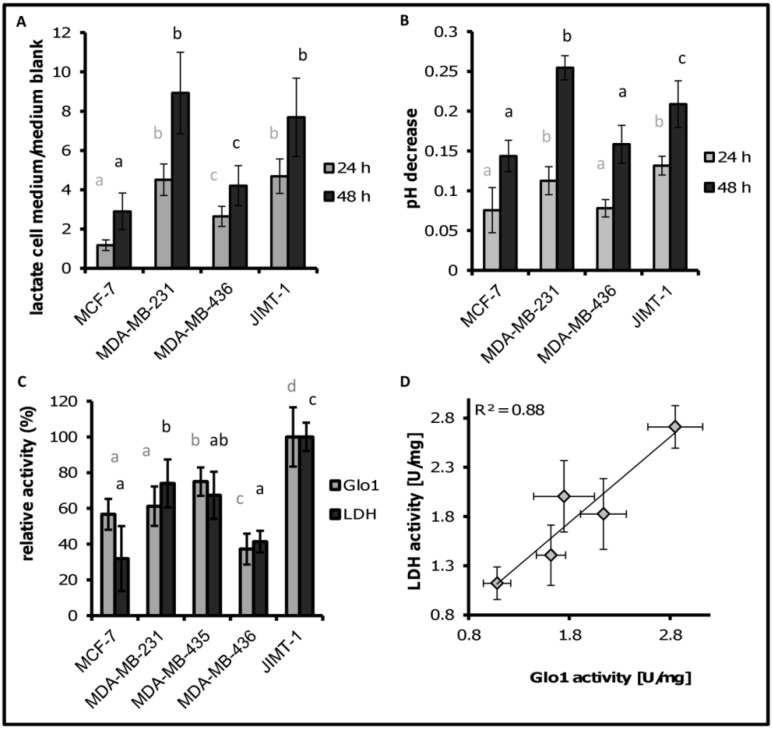
(**A**) Lactate release for the cell lines. Signal ratios were calculated by dividing signals from medium samples with cells by the signal of medium blank measurements; (**B**) pH difference between medium blank and cultivation medium after 24 h and 48 h; (**C**) maximum normalized enzyme activities for LDH and Glo1 for the five cell lines; same letters indicate no significant difference, different letters = significant difference between cells with *p <* 0.05. n = 6 for lactic acid release, pH value and Glo1 activity, n = 4 for LDH activity; and (**D**) Correlation between LDH activity and Glo1 activity in cells.

Consequently, we measured metabolites and pH decrease of the cell medium after cultivation for 24 h and 48 h (Figure 2A). After 48 h, lactate release of MDA-MB-231 was more than three times higher than for MCF-7 cells. The invasive cell line JIMT-1 also had a comparably high lactate release while the less invasive cell line MDA-MB-436 exhibited lower relative lactate content after 48 h. The pattern of lactate release was mirrored by a decrease in pH in the cultivation medium during the same time period (Figure 2B).

These results suggest that (i) the investigated cell lines may have different metabolic fluxes through glycolysis, which is (ii) not necessarily reflected by concentrations of the intracellular glycolytic metabolites.

### 3.3. LDH and Glo1 Activity Correlated with Extracellular Lactate Concentration

We assessed activities of enzymes related to glycolysis to further investigate the differential pattern of glycolytic flux among the cell lines, suggested by differential lactate concentration in the medium. In addition to hints on higher activity of PK observed with higher glycolytic activity (data not shown), LDH and Glo1 enzyme activities exhibited a similar pattern compared to the observed lactate release (Figure 2C). LDH catalyzes the interconversion of pyruvate and lactate, and the majority of extracellular lactate released upon glycolytic activity was shown to be produced from the conversion of pyruvate to lactate by LDH [43]. In agreement, LDH activity resembled the extracellular lactate pattern of the cell lines (Figure 2C). The relative activity of LDH in MCF-7 cells was lowest, followed by MDA-MB-436 cells that showed also relatively low lactate release compared to MDA-MB-231 and JIMT-1. Both cell lines with significantly higher relative activity of LDH also showed significantly higher lactate content in the cultivation medium (Figure 2A).

We additionally determined Glo1 activity as an enzyme whose activity is assumed to be related to the glycolytic shift of tumor cells [15]. The glyoxalase system consisting of Glo1 and Glo2 is the main cellular detoxification system for methylglyoxal (MGO), a toxic by-product of glycolytic glucose degradation [14]. Glo1 catalyzes the rate-limiting step in a two enzyme-system for the conversion of MGO to lactate. If the glycolytic activity in tumor cells increases, Glo1 activity should also increase to prevent cellular damage [15]. We found the highest Glo1 activity in JIMT-1 cells, while the tumor cell line MDA-MB-436 showed the lowest Glo1 activity followed by MCF-7. The differences between the mean values of Glo1 activity for each cell line were always significant (*p <* 0.05) as indicated in Figure 2C. The relative enzyme activity pattern of LDH correlated with the pattern of Glo1 (r = 0.94, *p* = 0.023, Figure 2D).

This suggests that Glo1 activity may indeed be enhanced in relation to glycolytic activity in agreement with the assumption that enhanced Glo1 activity is needed for detoxification from toxic by-products of this process. Upregulation of Glo1 in cancer cells was shown previously in various cancer tissues including breast cancer [15,44,45]. Bair *et al.* [15] found an increase in MGO-derived protein adducts after reduction of cellular Glo1 content using siRNA. However, although it was shown recently that Glo1 may be regulated in response to redox-relevant processes [46], a correlation of Glo1 enzyme activity with lactate release, and therefore with the glycolytic activity of breast cancer cell lines in particular, has not yet been proposed.

The relation of LDH and glycolysis and the regulation of LDH as one of the enzymes controlling the switch between glycolysis and oxidative phosphorylation [47] is well established in the literature [48]. In contrast, although it is widely accepted that Glo1 expression is high in cancer tissue and cell lines [15,49], the regulation of Glo1 activity is not well documented, only few authors reported on this [15,46]. For cellular mRNA expression analyzed by PCR, a similar pattern to cellular protein content was observed (see the Supplementary Figure S1C). Differences of relative intensities were higher especially for MCF-7 and MDA-MB-231. In return, the pattern of Glo1 enzyme activity in the five cell lines was similar to the pattern of Glo1 cellular protein content determined by western blotting (see the Supplementary Figure S1B) and a linear correlation was confirmed (r = 0.67, *p* = 0.006, n = 15). These results suggest differential regulation of Glo1 on mRNA expression rather than on protein level between the cell lines.

### 3.4. Cell Migration Correlates with Extracellular Lactate Concentrations, LDH and Glo1 Activity, and Glycerophosphoglycerol, β-Alanine and Pantothenic Acid

Beside its relation to glycolytic activity [42], lactate released to the extracellular space of cancer cells was also found to promote cell migration through acidification, which was associated with the metastatic potential of tumors [50]. The production of extracellular lactate of the cell lines under investigation is in agreement with known data on metastatic potential (Table 1) as MDA-MB-231 and JIMT-1 are highly invasive. Consequently, we assessed migration behavior in relation to lactate release in this study. To this end, cell migration of the five cell lines was determined using the Boyden chamber assay. A significant positive correlation of the number of migrated cells was found with lactate release, as well as with Glo1 and LDH activity (Table 3).

Cell migration was previously found to be related to the glycolytic flux of tumor cells [51]. Recently, Cheng *et al.* [18] found a correlation between Glo1 expression and tumor cell migration in gastric cancer indicating that increased Glo1 expression may be involved in processes enhancing cell migration. Further, they found significantly enhanced Glo1 expression in late stage tumors (T3/T4) and tumors with lymph node metastasis. Glo1 activity was also shown to be particularly increased in the more aggressive and invasive forms of tumor cells, for example, in ovarian cancer [16] and in Her2/neu overexpressing breast cancer [52].

To confirm the observation that Glo1 activity was indeed positively correlated to cell migration, we measured cell migration of a previously created stably transfected Glo1 knockdown clone (siGlo1, <10% Glo1 mRNA compared to the control) using MCF-7 as wild-type cells (MCF-7 wt). Cell migration, lactate release and LDH activity were also significantly decreased in siGlo1 cells while no difference could be observed in a mock transfected control (data not shown). Taken together, we found a comparably high glycolytic flux combined with high cell migration in the three cell lines MDA-MB-231, MDA-MB-435 and JIMT-1, while MCF-7 and MDA-MB-436 showed low lactic acid release and also lower cell migration.

Knowing that Glo1 and LDH activity as well as cell migration coincide with lactate release, we wanted to test whether relative intensities of any intracellular metabolites would also correlate with the glycolytic activity in the cell lines. Therefore, Pearson correlation coefficients and significance levels were calculated for both sample sets between all detected metabolites and lactate release, Glo1 and LDH activity and cell migration. Table 3 shows the Pearson correlation coefficients and the significance levels for the three metabolites glycerophosphoglycerol (GPG), pantothenic acid (PA) and beta-alanine. Significant positive correlations are displayed with the parameters related to tumor aggressiveness investigated here, namely lactate release, LDH and Glo1 activity and cell migration in both sample sets. The three metabolites listed in Table 3 were the only ones reproduced in both sets emphasizing the importance of independent replicate set measurements in metabolomic analysis.

**Table 3:** Pearson’s correlation between extracellular lactate, Glo1 and LDH activity, number of migrated cells (migration) and relative metabolite intensities for the five breast cancer cell lines (sample set 1), and for the breast cancer cell lines and an Glo1 knockdown mutant MCF-7siGlo1 and the corresponding mock transfected control cells of the cell line MCF-7, MCF-7 mock (sample set 2). The dataset was calculated on basis of mean values, n = 6 for the metabolite profiles and Glo1 activity per cell line, n = 5 for cell migration, n = 4 for LDH activity.

	Sample set	extracellular lactate*		Glo1 activity		LDH activity		Cell migration	
		r	p	r	p	r	p	r	p
Glycerophosphoglycerol (5TMS)	2	0.77	0.009	0.87	0.001	0.91	9.50×10^−5^	0.98	5.03×10^−7^
	1	0.81	0.085	0.88	0.016	0.95	0.002	0.78	0.057
Pantothenic acid D (3TMS)	2	0.87	0.001	0.84	0.001	0.85	0.001	0.86	0.001
	1	0.67	0.282	0.91	0.007	0.85	0.026	0.95	0.002
Alanine, beta- (3TMS)	2	0.91	1.24×10^−4^	0.63	0.069	0.82	0.003	0.82	0.003
	1	0.99	2.38×10^−4^	0.63	0.204	0.85	0.023	0.63	0.206
extracellular lactate	2			0.84	0.002	0.80	0.004	0.85	0.001
	1			0.72	0.104	0.89	0.013	0.82	0.037
Glo1 activity	2	0.84	0.002			0.76	0.010	0.86	0.001
	1	0.72	0.197			0.94	0.004	0.86	0.020
LDH activity	2	0.80	0.004	0.76	0.010			0.92	9.25×10^−5^
	1	0.89	0.029	0.94	0.004			0.81	0.040
Cell migration	2	0.85	0.001	0.86	0.001	0.92	9.25×10^−5^		
	1	0.82	0.078	0.86	0.020	0.81	0.040		

*extracellular lactate was not analyzed for MDA-MB-435, therefore n = 4 for this correlation using values from sample set 1.

Beta-alanine is a direct precursor of PA which is needed for the synthesis of coenzyme A (CoA). CoA acts as an acyl group carrier to form acetyl-CoA and other related compounds as a means to transport carbon atoms within the cell. Further, in the TCA cycle, CoA is important for pyruvate to enter as acetyl-CoA, and for α-ketoglutarate to be transformed to succinyl-CoA. CoA is involved in the biosynthesis of many important compounds such as fatty acids, cholesterol, and acetylcholine.

According to KEGG pathways, PA and GPG are not in the direct “neighborhood” of glycolysis or the TCA cycle. Slyshenkov and colleagues [53] showed protective effects of PA in tumor cells against lipid peroxidation and suggested that this is due to increased production of CoA, an essential enzymatic cofactor in phospholipid synthesis rather than quenching of free radicals. Furthermore, GPG, the second metabolite following the glycolytic pattern, is a main precursor in phospholipid synthesis. It was suggested that increased CoA level could promote cellular repair mechanisms and potentiate synthesis of membrane phospholipids [54]. PA was further found to prevent cells from damaging effects by providing increased concentrations of GSH in cells [55]. Since GSH is an essential cofactor in the glyoxalase system and CoA is obligatory in phospholipid biosynthesis, PA could indirectly promote membrane repair processes and removal of lipid peroxidation products by increasing cellular GSH concentration [53]. Various studies report a change in the phospholipid spectrum during tumor cell progression and an influence of specific phospholipids on tumor cell migration [26,56]. For example, phosphatidic acid, the simplest form of glycerophospholipids, can potentiate migration of invasive breast cancer cell lines even in nanomolar concentration [56]. Within this context, Mashima *et al.* [28] showed that highly proliferating cancer cells must synthesize fatty acids *de novo* to continually provide lipids for membrane production; synthesized fatty acids are used for energy production through β-oxidation and for lipid modification of proteins [28].

Thus, in summary we suggest higher concentrations of extracellular lactate, beta-alanine and PA and enhanced LDH and Glo1 activity to be a response to enhanced aerobic glycolysis in cancer cells, while GPG concentration may be associated with enhanced cell migration. We found that this was reflected in comparisons of tumor cell lines with different aggressiveness. Cancer cell lines exhibiting different intensities of one or the other parameter can therefore be a valuable resource to study the interconnection and, potentially, the regulation of these parameters.

## 4. Conclusions

Non-targeted metabolite analysis identified significant differences between the breast cancer cell lines under investigation. The metabolite profile of MCF-7 as the only luminal-type and less invasive cell line, differed the most from the four other cell lines tested. Our results emphasize the potential of metabolite profiling as a non-targeted approach for classification of known cancer subtypes. We further show that a higher abundance of metabolites in a tumor cell line compared to a non-tumor cell line is not necessarily reproduced comparing highly tumorigenic cell lines to low tumorigenic ones, though it is highly suggestive. Concluding from this, metabolites with different concentrations in comparison of a non-tumorigenic with a tumorigenic cell line could also be specific for the used cell line only without general relevance as tumor markers. Within this context, the genetic subtype of the cancer cell line might be a particularly critical parameter.

Considering our results on metabolic changes with known relevance to tumorigenesis, we found enhanced glycolytic activity as indicated by lactate release accompanied by increased LDH and Glo1 enzyme activity. In addition, glycolytic activity in the cancer cell lines was associated with their migration behavior. To our knowledge, this is the first report on a relation between LDH, Glo1 activity and cell migration in human breast cancer cell lines. We found higher Glo1 and LDH activity to be associated with enhanced glycolytic activity and cell migration and, therefore, with tumor aggressiveness.

By correlating metabolite profiles to lactate release, Glo1 and LDH activity, and cell migration, we identified beta-alanine, pantothenic acid and glycerophosphoglycerol as potentially important metabolites of malignancy. These particular metabolites have not been associated to glycolytic activity or cancer cell migration in any previous study.

## Supplementary Files

Supplementary File 1 Supplementary Files (DOCX, 189 KB)
