# Supplementary material for: Glycerophosphoglycerol, Beta-Alanine, and Pantothenic Acid as Metabolic Companions of Glycolytic Activity and Cell Migration in Breast Cancer Cell Lines
Source: Metabolites. 2013 Nov 27;3(4):1084–101. doi: 10.3390/metabo3041084 (PMC3937838; doi:10.3390/metabo3041084)
Supplement: Supplementary File 1 — Supplementary Files (DOCX, 189 KB) [file metabolites-03-01084-s001.docx]

**Supplementary Files**

**Table S1.** Mean Ratios of Significant Different Metabolites between MCF-7 and the Other Cell Lines. Mean ratios (mean value of MCF-7/mean value other cell line) of metabolites that differ between the investigated cell lines and the reference cell line MCF-7 were calculated from the dataset including interday biological variation. All metabolites listed were confirmed in the reference dataset with a higher significance.

| **Metabolite** | **Fold change against MCF-7** | | | |  |
| --- | --- | --- | --- | --- | --- |
|  | **MDA-MB-231** | **MDA-MB-435** | **MDA-MB-436** | **JIMT-1** | **Pathway according to KEGG [37]** |
| Alanine (2TMS) | 0.57 | 0.46* | 0.37** | 0.17*** | Ala/ asp/ glu metabolism |
| Glutamic acid, DL- (3TMS) | 0.34* | 0.47* | 0.31*** | 0.26*** | Ala/ asp/ glu metabolism |
| Proline, 4-hydroxy DL-trans (3TMS) | 26.94* | 39.68* | 15.38* | 5.30* | Arg/ pro metabolism |
| Ornithine, DL- (4TMS) | 6.32* | 3.00 | 1.23 | 1.40 | Arg/ pro metabolism |
| Putrescine (4TMS) | 3.03* | 3.54* | 0.78 | 0.35** | Arg/ pro metabolism |
| Creatinine (2TMS) | 2.10 | 3.47 | 0.20* | 0.06* | Arg/ pro metabolism |
| Methionine, DL- (2TMS) | 1.99 | 1.57 | 0.57 | 0.29*** | Cys/ meth metabolism |
| Threonine, DL -(3TMS) | 0.37 | 0.34** | 0.12** | 0.09* | Gly/ ser/ threo metabolism |
| Butyric acid, 4-amino (3TMS) | 0.06 | 0.05** | 0.03** | 0.10* | Gly/ ser/ threo metabolism |
| Tyrosine, DL- (2TMS) | 0.51 | 0.71 | 0.23 | 0.08** | Phe/ tyr/ trp metabolism |
| Valine, DL- (2TMS) | 0.54 | 0.41* | 0.29** | 0.13*** | Val/ leu/ ile metabolism |
| Octadecan-1-ol, n- (1TMS) | 30.54* | 55.68* | 36.51* | 47.16* | Fatty acid metabolism |
| Hexadecan-1-ol, n- (1TMS) | 19.21* | 15.19* | 25.19* | 15.75* | Fatty acid metabolism |
| Inositol myo (6TMS) | 0.17*** | 0.18*** | 0.27*** | 0.27*** | Galactose metabolism |
| Glucose-6-phosphate (1MEOX)(6TMS) | 13.81** | 8.40** | 2.40* | 2.20 | Glycolyse/ gluconeogenese |
| Lactate, DL- (2TMS) intracellular | 0.42*** | 0.48* | 0.34*** | 0.44*** | Glycolyse/ gluconeogenese |
| Pantothenic acid, D- (3TMS) | 0.19*** | 0.04** | 0.44 | 0.02* | Pantothenate and CoA biosynthesis |

*p < 0.05; **p < 0.01; ***p < 0.001

**Table S2.** Abundance of Metabolites Belonging to TCA Cycle or Glycolysis Detected in the Five Breast Cancer Cell Lines under Investigation and Related to MCF-7 as a Reference. Analysis of intracellular metabolites; detected compounds belonging to TCA cycle or glycolysis are displayed. Relative abundance of each analyte compared to the abundance in MCF-7 as reference cell line (mean value MCF-7/mean value other cell line) are listed for sample set 1 if the metabolite was found in the dataset including biological variation, sample set 1 and 2 if the results obtained in both sample sets were contradictory, or in sample set 2 if the analyte was only detected there. Bold numbers show values that meet the required criteria according to [29] (mean ratio >2, *p* < 0.05), n.a. = not analyzed.

| metabolite | pathway | Sample set | MDA-MB- 231 | | MDA-MB-435 | MDA-MB-436 | | JIMT-1 |
| --- | --- | --- | --- | --- | --- | --- | --- | --- |
|  |  |  | Fold change against MCF-7 | | | | | |
| Oxalic acid (2TMS) | TCA-cycle | 1 | 0.2 | 1.7 | | | 1.9 | 0.3 |
| Citric acid (4TMS) | TCA-cycle | 1 | 2.1 | 1 | | | 1.9* | 1.3 |
| Succinic acid (2TMS) | TCA-cycle | 1 | 1 | 0.7 | | | 0.5* | 0.6* |
| Malic acid DL (3TMS) | TCA-cycle | 1 | 0.6 | 0.7 | | | 0.2* | 0.5* |
| Fumaric acid (2TMS) | TCA-cycle | 1 | 0.4 | 0.7 | | | 0.1* | 0.7 |
| Glutaric acid, 2-oxo (1MEOX)(2TMS) | TCA-cycle | 2 | 0.34* | n.a. | | | 1.16** | 0.26** |
| Glucose D (1MEOX)(5TMS) | glycolysis | 1 | 6.5*** | 3.3*** | | | 2.3*** | 1.6 |
|  |  | 2 | 0.98 | n.a. | | | 0.9 | 0.9 |
| Glucose-6-phosphate (1MEOX)(6TMS) | glycolysis | 1 | 14** | 8.4** | | | 2.4* | 2.2 |
|  |  | 2 | 0.5 | n.a. | | | 0.5 | 0.9 |
| Fructose D (1MEOX)(5TMS) | glycolysis | 1 | 4.2* | 0.8 | | | 0.5 | 1.5 |
|  |  | 2 | 0.88 | n.a. | | | 0.5*** | 0.9 |
| Fructose-6-phosphate (1MEOX) (6TMS) | glycolysis | 1 | 4.8* | 4.8* | | | 1 | 1.2 |
|  |  | 2 | 0.4*** | n.a. | | | 0.5*** | 0.8** |
| Dihydroxyacetone phosphate (1MEOX)(3TMS) | glycolysis | 2 | 0.3** | n.a. | | | 0.6* | 0.5** |
| Glycerol-3-phosphate DL (4TMS) | glycolysis | 1 | 1.2 | 1 | | | 0.7 | 0.7 |
| Glycerol-2-phosphate (4TMS) | glycolysis | 1 | 1 | 0.7 | | | 0.3 | 0.8 |
| Glyceric acid-3-phosphate (4TMS) | glycolysis | 1 | 1 | 1.1 | | | 0.7 | 0.6 |
| Glyceric acid L (3TMS) | glycolysis | 1 | n.d. | n.a. | | | 1.2 | 0.5 |
| Lactate DL (2TMS) | glycolysis | 1 | 0.4*** | 0.5*** | | | 0.3*** | 0.4*** |
|  |  | 2 | 0.57* | n.a. | | | 1.25 | 0.55* |
| Pyruvate (1MEOX) (1TMS) | glycolysis | 1 | 0.7 | 0.9 | | | 0.7 | 1.3 |

**p* < 0.05; ***p* < 0.01; ****p* < 0.001.

**Figure S1.** Relation between Glo1 mRNA Expression, Protein Amount and Enzyme Activity. (**A**) Relative Glo1 mRNA expression of the five breast cancer cell lines assessed using semiquantitative PCR with β-actin as reference gene; (**B**) Glo1 protein content determined by western blotting using polyclonal antibodies against Glo1, each lane contains 40 µg protein; and (**C**) Relative intensity of Glo1mRNA (n = 6) assessed with RT-qPCR using HMBS as reference; relative protein content was evaluated by densitometric immunoblotting and related to cellular protein content of β-actin (n = 3) and relative Glo1 enzyme activity (n = 6, **p* = 0.05; ***p* = 0.01; ****p* = 0.001).

**
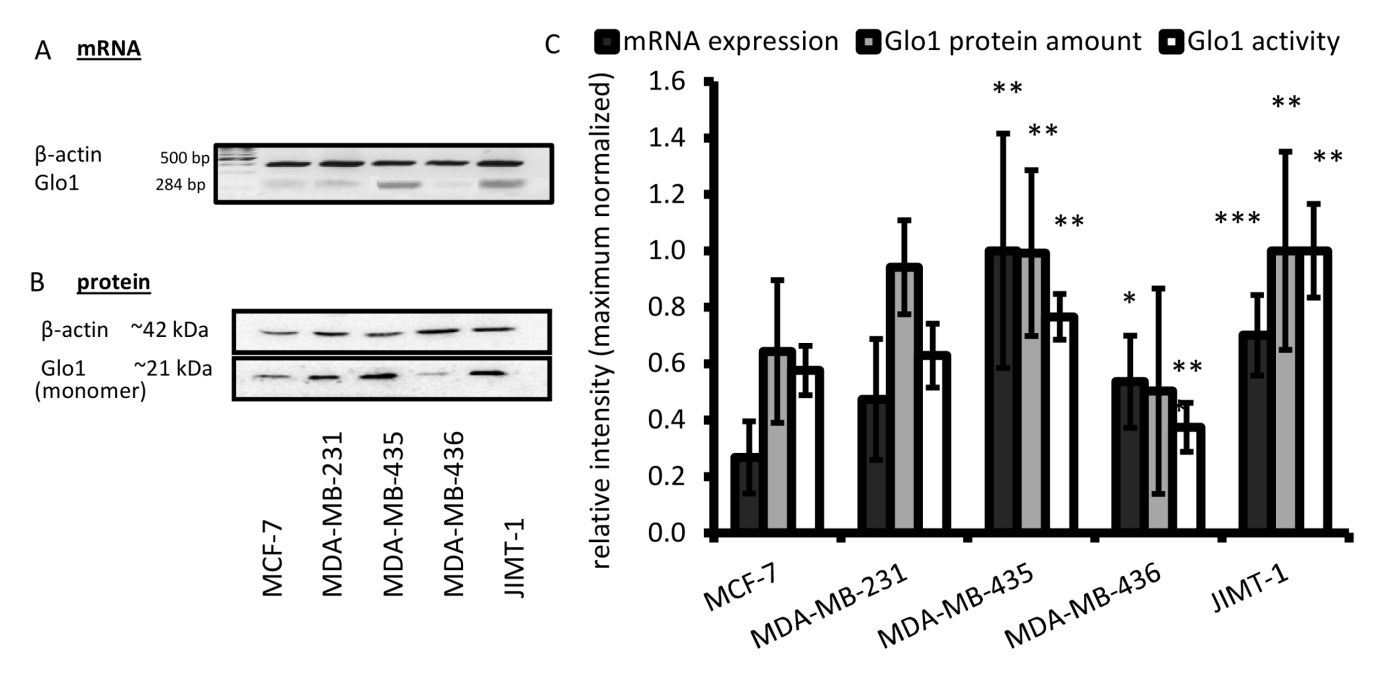
**
